# Non-contact characterization of compound optical elements using reflectance confocal microscopy, low-coherence interferometry, and computational ray-tracing

**DOI:** 10.1038/s41598-019-53369-x

**Published:** 2019-11-19

**Authors:** Mohamed T. El-Haddad, Yuankai K. Tao

**Affiliations:** Department of Biomedical Engineering, Vanderbilt University, Nashville, TN USA

**Keywords:** Imaging and sensing, Microscopy, Optical spectroscopy

## Abstract

Advances in microscopy have enabled us to see at unprecedented depths and resolutions, even breaking the diffraction-limit by several fold. These improvements have come at the expense of system complexity with microscopes routinely employing multiple objective lenses and custom optical relays. Optimal system design is paramount for imaging performance, but research systems are limited by the use of commercial components because optical prescriptions are often inaccessible. System performance can be further degraded when these components are implemented in nonstandard configurations outside of manufacturer specifications. Here, we describe a method for characterization of compound optical elements including curvatures, material and air-gap thicknesses, and glass types. We present validation data for doublets and a commercial broadband scan lens. Our method is both non-contact and non-destructive, and we believe it addresses a unique gap in optical design that may be extended to broad applications in both research and industrial manufacturing.

## Introduction

The need for deeper and higher resolution optical imaging in biological research has led to advances in microscopy, including light-sheet^1–4^, nonlinear contrast^5–9^, and super-resolution^10–13^. However, with increasing system complexity, optical aberrations become more detrimental to imaging depth, resolution, and/or signal and must be carefully managed. In light-sheet microscopy, aberrated excitation increases sectioning thickness and reduces signal-to-background ratio by orders-of-magnitude while aberrated collection degrades lateral resolution^3,4^. Two-photon fluorescence^5^, second harmonic generation (SHG)^8^, and coherent anti-stokes Raman spectroscopy (CARS)^9^ signals vary nonlinearly with excitation intensity, and SHG and CARS are phase sensitive. Thus, aberrations in these systems may result in significant loss of both resolution and excitation efficiency. Finally, super-resolution techniques are particularly sensitive to aberrations because of the strict requirements on excitation point-spread-function shape^14–16^, use of high numerical aperture (NA) objectives, and need to resolve nanometer-scale features.

The aforementioned imaging systems often utilize commercial optical components in orientations, over wavelength regimes, across field-of-views (FOV), and/or with immersion media outside of design specifications, resulting in increased aberrations and suboptimal performance. Adaptive optics (AO) has been integrated with microscopy systems to correct for the combination of system and sample-induced aberrations^16–19^. The utility of AO is offset by the need for a complex and expensive imaging system with minimal aberrations because only a limited range of wavefront errors can be corrected. Unfortunately, optimal system design and simulation is often impossible because prescription data for many commercial optics are not publicly available. Thus, there is a need for non-destructive methods to characterize prescriptions of multi-element optical components.

Optical elements can be characterized using either wavefront sensing^20^ or optical ray-tracing^21–23^ methods to obtain an aberration map or ray-transfer matrix, respectively. Aberration maps for unknown optical elements may be used to drive designs and optimizers that minimize cumulative system aberrations. However, these aberration maps would need to be measured at each operating wavelength and incidence angle to comprehensively simulate optical performance. While useful for simulating light propagation in optical systems, ray-transfer matrices also require exhaustive spectral and spatial characterization, are only valid in the paraxial approximation, and provide no phase information. Estimation of the prescription data of unknown lens elements precludes the need for exhaustive physical measurements and may be directly integrated with industry-standard optical design and simulation software packages.

Low-coherence interferometry (LCI) provides coherence-gated imaging in transparent and semi-transparent media with up to 1 μm axial resolution and over 100 dB sensitivity^24^. Application of LCI in metrology have included surface profilometry^25–27^, measurement of internal thicknesses of optical assemblies^28^, and estimation of internal curvatures^27,29^. However, accurate reconstruction of these sample geometries requires *a priori* knowledge of material properties because path-lengths measured using LCI are scaled by the group velocity of the illumination in the propagation medium. In unknown materials, several groups have demonstrated simultaneous thickness and refractive index measurements using “focus-tracking” to quantify focal plane shifts within the sample^30–36^. Many of these previous methods relied on approximating the phase (n_p_) and group (n_g_) refractive index, which limited measurement accuracy. n_p_ and n_g_ may be decoupled by estimating the dispersion parameter, but this typically requires measurements in transmission and assumes a homogenous sample, which is not suitable for characterization of compound optical elements^36–42^.

Here, we present a method for characterization of compound optical elements using LCI, reflectance confocal microscopy (RCM), and computational ray-tracing. We demonstrate measurement of external and internal surface radii, internal glass and air-gap thicknesses, and estimation of glass materials. We validate the method on a set of 6 commercial achromatic doublet lenses and characterize a commercial scan lens. We believe this novel method overcomes unique barriers in the design of optical imaging systems and manufacturing of precision optical components.

## Results

### External radius measurement

**Figure 1:**
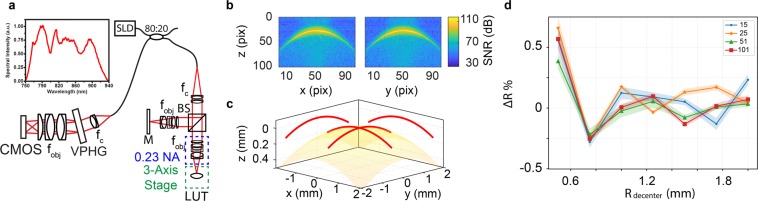
System schematic and calibration data. (**a**) 20% of the laser source was relayed to a free-space Michelson interferometer with 0.23 image space NA (S1). The back-coupled signal was detected using a custom-built spectrometer. (**b**) Representative cross-sections of a ceramic reference sphere surface sampled out to ±1.5 mm (R_decenter_) showing loss of specular reflection signal with decenter. (**c**) Segmented lens surface converted from pixels to physical units and fit using a best-fit sphere. (**d**) Percent deviation between measured and manufacturer specification for reference sphere radius at different sampling densities (number of samples per cross-section). Data points and shaded regions represent mean percent error and standard deviation, respectively, (n = 5). BS, cube beamsplitter; CMOS, line-scan camera; f, lenses; SLD, super luminescent diode; VPHG, volume phase holographic grating.

LCI measurements of external lens surfaces were performed using a broadband free-space Michelson interferometer detected on a custom-built spectrometer (Fig. 1(a)). System sensitivity was 96.2 dB for 720 µW incident power at the lens-under-test (LUT) and 3.5 µs spectrometer exposure time. LCI axial resolution was 2.17 µm in air and a 0.23 NA sample objective was used for epi-illumination and collection. Aberrations and field distortions in the system were minimized by translating the LUT relative to a stationary focus using a 3-axis motorized stage assembly. External lens surfaces were sampled along orthogonal (x- and y-) axes, and these depth cross-sections of the lens surface (Fig. 1(b)) were segmented using the peak positions of each axial reflectivity profile. The resulting point cloud was fit to a sphere by least-squares minimization to obtain the lens external radius and center of curvature.

A ceramic reference sphere with 14.9561 mm diameter and 300 nm tolerance (RS-DK15-C, Carl Zeiss Industrial Metrology, LLC, USA) was imaged, segmented, and used to calibrate axial dimensions in LCI depth cross-sections (Fig. 1(b,c)). LCI external radius measurement accuracy was evaluated by imaging the reference sphere out to various decentered positions (R_decenter_) and with various sampling densities. Figure 1(d) shows that external radius measurement accuracies converge to ±0.2% of the manufacturer specification. As expected, measurement accuracy was dominated by R_decenter_ and not sampling density because, in the ideal case, a spherical surface geometry can be fit with as few as three samples.

The relationship between external radius measurement accuracy and decenter at which the measurements are taken is confounded by two practical limitations. First, the surface reflectivity decreases with increasing decenter as a function of collection NA. Low signal results in surface segmentation ambiguity and errors in sphere fitting. Second, external surface measurements are of purely specular reflections and, thus, defocus and local surface slope lead to LCI path-length ambiguities due to mismatch between the incident and reflected beam paths. Therefore, LCI measurements include an additive error term that underestimates the external radius of curvature, which is proportional to both the measurement decenter position and NA. We minimize contributions from both of these aforementioned sources of error by using a signal-to-noise ratio (SNR) threshold to remove surface measurements prior to sphere fitting, which rejects measurements corresponding to local slopes exceeding approximately NA/2 (Supplementary Fig. S2 and S3). All measurements in this manuscript used an empirically determined SNR threshold of −27.5 dB relative to the maximum reflectivity at each measured surface.

**Figure 2:**
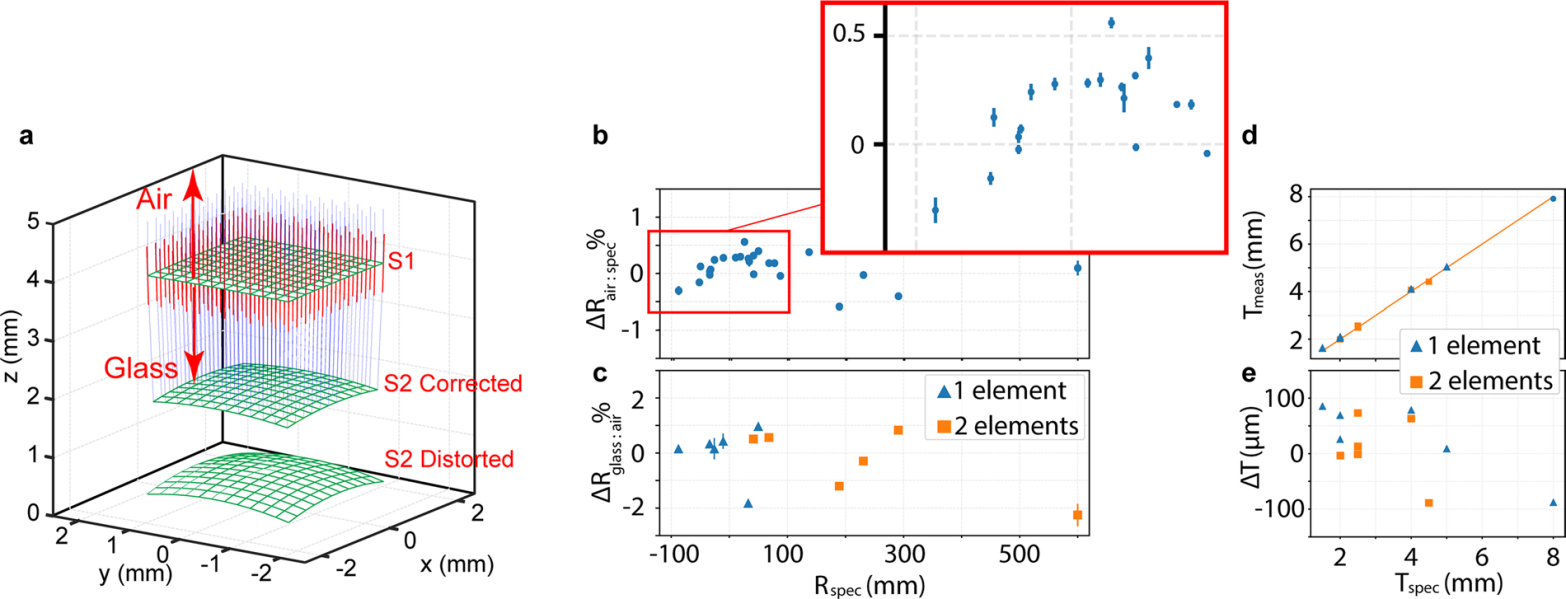
Characterization of multi-element lenses. (**a**) Computational ray-trace model for correction of internal surface curvatures. Blue lines represent simulated chief rays and red lines show the corresponding surface normal at the measured external lens surface (S1). S2 Distorted denotes the internal surface segmented from LCI measurements. Each chief ray is refracted at S1 by the phase index and propagated by its respective distance through the lens thickness, which is calculated by scaling the corresponding optical distance between S1 and S2 Distorted by the group index. The resulting three-dimensional propagated chief ray coordinates are used to remap S2 Distorted to a distortion-corrected internal surface (S2 Corrected). (**b**) Percent deviation between radius of curvature measured externally and their respective manufacturer specification (R_air:spec_). Inset shows a magnified view of the cluster of points between ±100 mm specification curvature. (**c**) Percent deviation of internally measured radius of curvature, through either 1 or 2 glass elements, relative to the corresponding externally measured curvature (R_glass:air_). Data points and error bars represent mean percent deviation and standard deviation, respectively (n = 5). (**d**) Plots of the measured thicknesses (T_meas_) and (**e**) corresponding errors relative to the manufacturer specification (T_spec_). Manufacturer tolerances were ±1% for radius and ±200 µm for thickness.

We validated this external radius measurement method on a total of 24 surfaces from 6 uncemented achromatic doublets with varying curvatures (Table 1). All errors in measured external radii were within ± 0.5% of manufacturer specifications and tolerances (Fig. 2(b)).

**Table 1:** Comparison between manufacturer specifications (gray rows) and measure parameters (white rows) for all characterized lens elements.

Lens		Glass	R1	R2	R3	T	Phase Index (n_p_) @ 830 nm	Abbe (v_d_)	SPHA	CLA	BFL
1	AC127-025-A	N-BAF10	18.8	−10.6	−68.1	5	1.6591	47.11	8.81	−1.90	21.39
		SF10				2	1.7099	28.53			
		H-ZBAF5	18.86	−10.72	−68.23	5.007	1.6601	47.28	9.33	−1.78	21.35
		ZF4				1.996	1.7098	28.32			
2	AC127-075-A	SF2	−137.1	−34.0	41.3	1.5	1.6336	33.82	0.14	−0.62	73.10
		N-BK7				2.5	1.5102	64.17			
		SF2	−137.62	−34.12	41.43	1.583	1.6336	33.82	0.16	−0.41	72.97
		S-APL				2.573	1.5112	69.56			
3	ACN254-050-A	N-BAF10	−34.0	32.5	189.2	2	1.6591	47.11	−16.42	3.95	−52.98
		N-SF6HT				4.5	1.7826	25.36			
		K-LaKn7	−33.99	31.99	188.09	2.067	1.6599	51.72	−16.09	2.59	−52.88
		Q-SF6S				4.411	1.7806	25.53			
4	ACN254-100-A	N-BAK4	−52.0	49.9	600.0	2	1.5608	55.97	−1.16	1.76	−103.88
		SF5				4	1.6574	32.25			
		H-BAK7GT	−51.92	50.58	600.95	2.024	1.5608	56.06	−1.42	3.06	−103.45
		BAH32				4.063	1.6572	39.28			
5	AC254-060-A	E-BAF11	41.7	−25.9	−230.7	8	1.6606	48.36	8.34	−2.28	54.15
		FD10				2.5	1.7097	28.32			
		H-ZBAF16	41.70	−26.0	−230.64	7.910	1.6561	48.43	10.25	−2.69	53.56
		P-SF69				2.499	1.7046	29.23			
6	AC254-200-A	N-SSK5	77.4	−87.6	291.1	4	1.6484	50.88	0.29	−0.54	194.31
		LAFN7				2.5	1.7335	34.95			
		H-ZBAF50	77.54	−87.47	289.93	4.0771	1.6484	50.87	0.31	−1.93	193.99
		H-LAF3B				2.513	1.7314	44.90			

Measurements for R1 and R3 were obtained in air while R2 measurements were performed through 1 glass element and corrected. Manufacturer tolerances were +/− 200 µm for thicknesses, 1% for radius, and +/− 1% for focal length. Spherical (SPHA) and chromatic (CLA) aberration and back focal length (BFL) for each lens were simulated in ZEMAX using a collimated polychromatic source spanning the laser source spectrum. The beam diameter was set to 95% of the full aperture and the focus was found by minimizing the root-mean-squared spot size in the image plane.

### Internal radius and thickness measurement

The high sensitivity of LCI enables detection of reflections from refractive index differences on the order of 10^−3^, making it uniquely suited for the evaluation of compound lens internal geometries. However, LCI measured internal curvatures are distorted by both refraction and path-length scaling, which are functions of the phase and group refractive index of lens elements, respectively. In addition, aforementioned radius of curvature accuracy considerations for external radius measurements are directly applicable to all internal surfaces. Here, we correct for distortions of internal lens geometries in post-processing by using optical properties of the respective lens glass material^29^.

A computational ray-trace model was used to simulate the LCI illumination optical paths for all measurement locations over the LUT sampling area (Fig. 2(a)). Chief rays incident on an initial surface (S1) were refracted and then propagated inside the glass material. Physical propagation distance was calculated using the difference between the LCI measured optical path-length between S1 and internal surface S2 Distorted. This difference was then scaled by the group index of the glass material evaluated at the LCI center wavelength. These simulated chief rays provided point-by-point three-dimensional coordinates for each distorted LCI measurement of the internal surface (S2 Distorted), which were used to remap the internal surface to a distortion-corrected internal surface (S2 Corrected).

Propagation and refraction of chief rays through upstream lens elements results in an aspheric distortion-corrected internal surface. Experimentally, this aspheric distortion-corrected internal surface sag (*Z*) was fit using even powers of the radial distance from the optical axis (*r*)^43^ with the vertex radius (*R*) corresponding to the radius of curvature of the actual spherical surface.

1 $$Z=\mathop{\sum }\limits_{n=1}^{8}{a}_{n}{r}^{2n}$$

2 $$R=\frac{1}{2{a}_{1}}$$

Internal radius measurements were validated on 12 surfaces from uncemented achromatic doublets (Table 1). Six surfaces were each imaged through one upstream lens element (i.e., back surface of a singlet measured through the lens, simulating internal surface of a doublet) and six were imaged through two upstream lens elements (i.e., back surface of a doublet measured through both singlets, simulating internal surface of a triplet). Percent deviation of the internal radius of curvature relative to the corresponding curvature measured in air (i.e., as external surfaces) was calculated to evaluate internal radius measurement accuracy without bias from manufacturing tolerances (Fig. 2(b,c)).

Lens element thicknesses were measured along the optical axis. The physical thickness was calculated by scaling the measured optical path-length between S1 and S2 Distorted by the group refractive index of the LUT materials (Fig. 2(d,e)).

### Glass material identification

**Figure 3:**
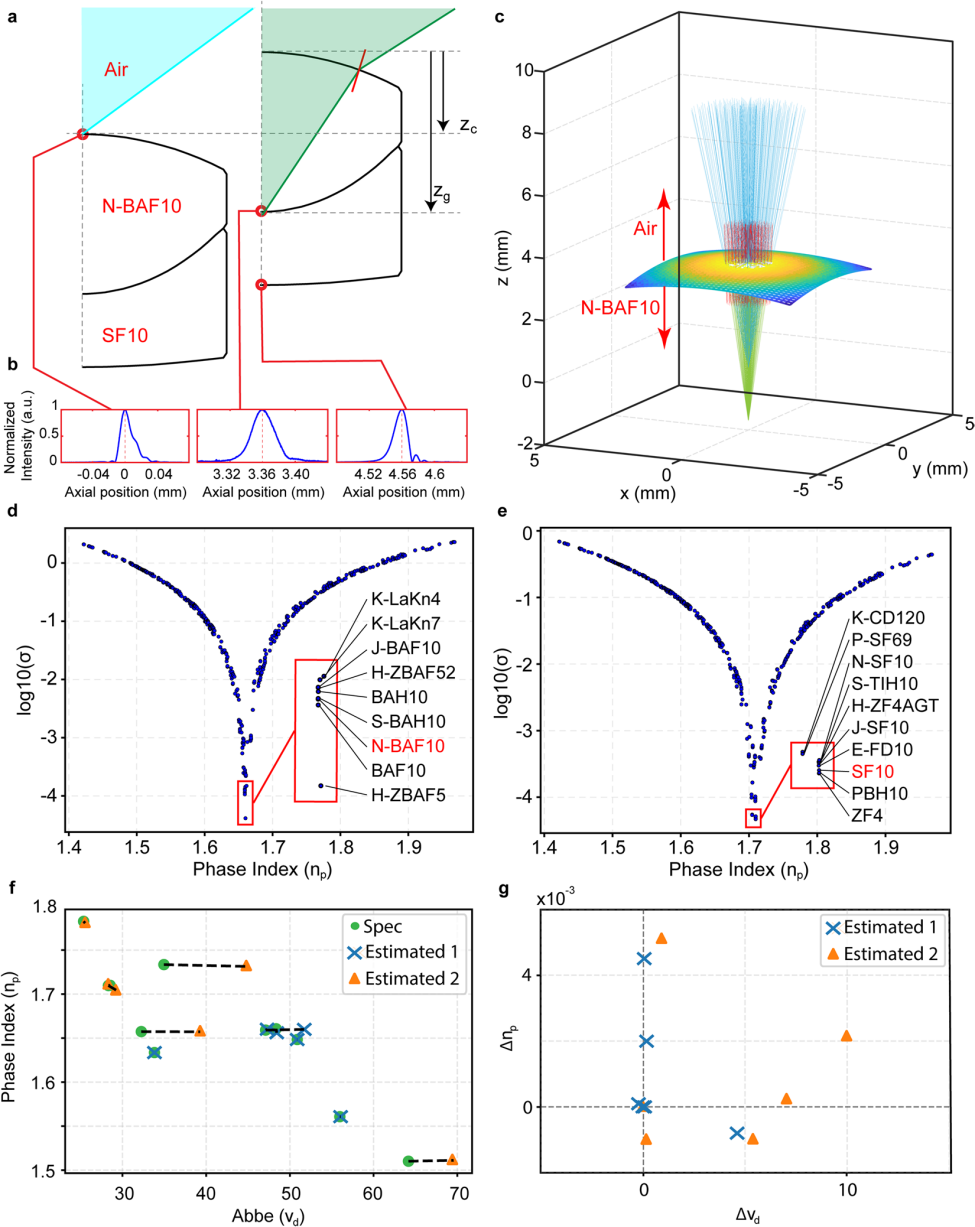
Glass material identification. (**a**) Geometric ray-trace model of RCM measurements showing the illumination cone focused on the front surface (blue), followed by the back surface (green) of the first lens element after the lens is translated axially by an offset (z_c_). z_c_ is the confocal thickness and differs from the glass element thickness (z_g_) due to refraction of the marginal rays of the illumination cone. The red line represents the front surface normal at its intersection with the marginal ray. (**b**) Representative on-axis RCM axial response measured at 3 surfaces of an achromatic doublet (AC127-025-A, Thorlabs, Inc., USA). (**c**) Corresponding computational ray-trace simulating RCM illumination. (**d**,**e**) Log-error plots corresponding to the glass elements in (**a**). The insets show magnified cluster of glass materials near the minima. Red labels show manufacturer specified glass material for each lens element in (**a**). (**f**) Phase index (n_p_) and Abbe number (v_d_) plot showing the relationship between identified and manufacturer specified glass material properties (dotted lines) for all 12 elements from Table 1. (**g**) Deviations in np and v_d_ of identified glass materials relative to manufacturer specifications. Estimated 1 and 2 indicate materials that were characterized through air and an upstream element, respectively.

Characterization of internal lens geometries relies on prior knowledge of glass material properties (i.e., group and phase index). We developed a novel method to identify unknown glass materials by using a combination of RCM and LCI measurements and modeling (Fig. 3(a)). RCM measurements were acquired by blocking the LCI reference reflector. The single-mode core of the fiber coupler acted as the confocal pinhole, which provided an RCM axial resolution of 16 *μm* at the center wavelength. The LUT was translated to measure the on-axis RCM axial response at the front and back surfaces of each optical element (Fig. 3(b)). The focal position at each surface was defined as the axial center-of-mass of the maximum 5% of RCM axial response intensities evaluated at the center wavelength. The axial difference between the focal positions of the two measured surfaces was defined as the confocal thickness (Fig. 3(a), z_c_), which was a function of both the glass material properties and lens geometry.

Glass material identification ambiguity was reduced by repeating LCI measurements of lens optical thickness along the optical axis at 3 wavelengths spanning the illumination spectrum (*λ* = 769, 830, 885 nm). This was achieved by adjusting the reference arm to center the zero-delay stationary phase point on each measurement wavelength^39^. These optical thicknesses included inherent information about material dispersion properties at each measurement wavelength.

A database of the optical properties of all commercially available glass materials defined within the illumination bandwidth (1340 materials) was compiled^44^. A computational ray-trace model of propagation through lens elements was then created to exhaustively simulate RCM measurements through each glass material (Fig. 3(c)). The axial focal positions of un-refracted (blue) and refracted (green) rays were computed using least-squares minimization of three-dimensional distances between the rays in the simulated illumination bundle. The unknown glass material was identified by minimizing the error function (*σ*) between simulated and measured parameters where,

3 $$\sigma ={(d-{d}_{s})}^{2}+2{(\Delta t-\Delta {t}_{s})}^{2},$$

4 $$\Delta {t}_{s}={d}_{s}({n}_{g,{\lambda }_{b}}-{n}_{g,{\lambda }_{e}})$$

5 $$d=\frac{t}{{n}_{g,{\lambda }_{o}}}$$

Subscript *s* denotes a simulated parameter; d and t denote geometric and optical thickness; and n_g_ denotes group refractive index evaluated at *λ*_*b*_, *λ*_*o*_, and *λ*_*e*_, which corresponds to wavelengths at the beginning, center, and end of the illumination bandwidth, respectively. Figure 3(d,e) show log(*σ*) plots for each material in the database as a function of phase index at *λ*_*o*_ for the two glass materials in Lens 1 (Table 1).

Glass material identification methods were also extended to multi-element lenses by performing RCM measurements and simulations serially through upstream elements. Identification accuracy was validated using 6 doublets (Table 1) by first identifying one optical element and then the second through the first. The phase index and Abbe numbers of the identified and manufacturer specified glass materials for all measured optical elements are plotted in Fig. 3(f) (dotted line denotes corresponding pairs of identified and specified material properties). Deviation of identified phase index and Abbe number relative to specification (Fig. 3(g)) shows our method is sometimes prone to overestimate of Abbe number, which is expected for spectroscopic measurements of optical properties using wavelengths in the near-infrared regime.

### Characterization of compound optical elements

**Figure 4:**
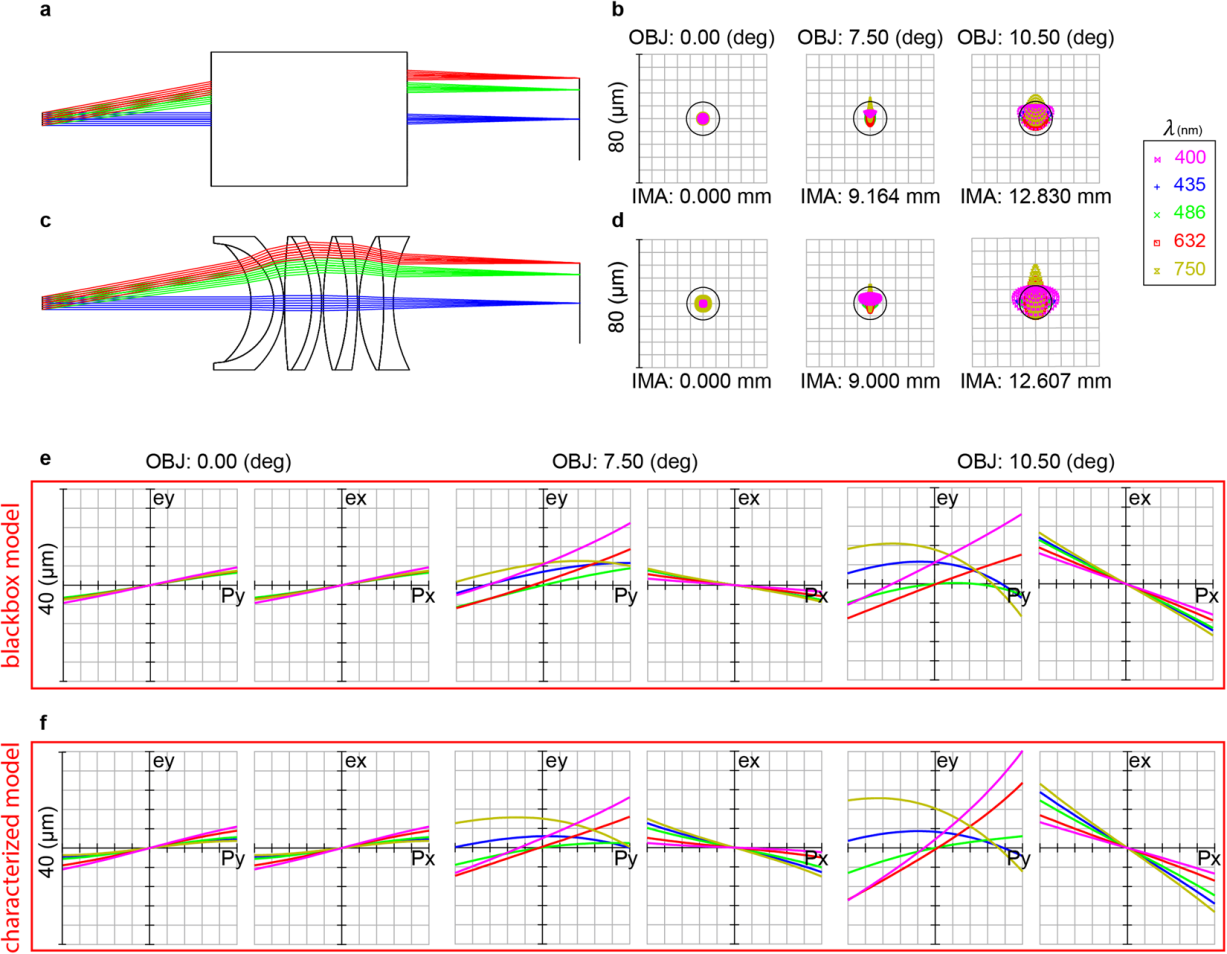
Ray-trace and comparison of optical performance. (**a**,**b**) Manufacturer black-box lens model and spot diagram for 0, 7.5, and 10.5 degree object space field angles. (**c**,**d**) Empirically measured prescription schematic, ray-trace, and spot diagram. Transverse ray aberration plots showing ray error over normalized pupil coordinates of (**e**) model vs (**f**) measured prescription at the design wavelengths.

We evaluated the robustness of our proposed method by characterizing a commercial broadband multi-element scan lens (CSL-SL, Thorlabs Inc., USA). The scan lens dimensions exceeded the working distance of our LCI/RCM system (19.5 mm), so measurements were acquired in both forward and backward directions and then combined to effectively double the available working distance. The optical performance of our empirically measured prescription data was compared against a black-box lens model provided by the manufacturer (Fig. 4).

An additional hammer optimization (OpticStudio, Zemax LLC, USA) for glass materials was performed on our empirically measured prescription data to minimize chromatic aberration arising from errors in the Abbe number (Fig. 3(g)). A subset of candidate glass materials was selected for each measured optical element from materials within 1 log(σ) of the optimum, within Abbe number and phase index ranges of ±10 and ±0.006, respectively. Our measured prescription data was then optimized for achromatic performance over the manufacturer specified operating wavelength range (Fig. 4, inset. Supplementary Fig. S4). The image space NA and effective focal length of our empirically measured prescription data were 0.0291 and 68.74 mm, respectively, as compared to 0.0286 and 70 mm in the black-box model. Spot diagram and image space centroid positions were used for qualitative comparison of the optical performance against the black-box model (Fig. 4(b,d)). Ray fan plots over the design scan angles and wavelengths were used to quantitatively compare transverse aberrations (Fig. 4(e,f)). These plots show that the black box and characterized models have near identical aberrations. We emphasize here that the differences between lens performance represent a combination of any characterization errors and inherent manufacturing tolerances.

## Discussion

LCI measurements of lens curvatures were performed using a free-space Michelson interferometer to ensure mechanical stability between reference and sample arms and minimize sub-coherence length vibrations (Supplementary Fig. S1). This is similar to previously reported observations in confocal microscopy profilometry of highly reflective surfaces^45^ in which the achievable accuracy was limited by sub-optical resolution surface features. Noise in the reported LCI measurements of surface curvatures was dominated by the accuracy of LUT translation, which may be improved significantly with higher-precision actuators. One limitation of our curvature measurement method was that a spherical geometry was assumed for all external and internal curvatures, which precludes modeling of higher-order surfaces. LCI may also be directly applied to tolerancing and characterization of aspheric, parabolic, and freeform surfaces, but these applications would require more robust data-fitting and ray-tracing methods.

The accuracy of optical element geometry measurements and glass material identification was dependent on LCI and RCM system specifications, namely NA, working distance, and illumination spectrum. These parameters may be directly tailored to specific applications and desired measurement accuracies. Non-contact lens characterization may also enable real-time feedback on surface geometries, thicknesses, centration, tilt, and axial spacing of optical elements to benefit real-time inspection in manufacturing. Glass materials are known *a priori* in these commercial applications and, thus, a lower NA LCI/RCM with longer working distance may be used to evaluate full cross-sections of large lens assemblies. Alternatively, submicron axial resolution measurement of surface curvatures may be achieved by use of ultrashort lasers sources. In research applications, such as biological microscopy, imaging systems routinely integrate commercial objectives. Full characterization and compensation of system aberrations require precise measurements of both glass material properties and lens geometries. Empirically measured prescription data of multi-element commercial objectives enables design optimizations for optical resolution and throughput.

Previous efforts to measure material optical properties were limited to either assuming flat slab geometries, only tracing the intersection between chief and marginal rays, and, in some cases, assuming an approximate equality between group and phase refractive index^30–36^. Our computational ray-tracing approach addresses all of these limitations and provides several key advantages that are well-suited for characterization of multi-element optical elements:

1. Computational simulation of an arbitrary number of rays more accurately simulates focal shift, inherently accounts for aberrations from both the system optics and LUT surfaces, and may be easily extended to arbitrary surface geometries.
2. Reducing glass material identification to an error minimization problem within the finite set of commercially available glasses constrains the solution-space and eliminates the need for approximate numerical solutions.
3. Spectroscopic measurements of optical thickness provide an indirect measure of dispersion, which further constrains the set of possible solutions for lens geometries and glass materials.

Aberrations for all characterized doublet lenses at the center wavelength of the illumination source were less than 2λ (mean: −0.32λ, std: 0.88λ) of the manufacturer specification (Table 1). Deviations in optical aberrations were primarily a result of errors in glass material identification, as further confirmed by the data obtained for the scan lens in Fig. 4. However, inaccurate glass estimates may also lead to errors in internal thickness and curvature measurements. For example, an error of δn_g_ in the estimated group refractive index of an element of thickness, t, leads to an error of −δn_g_t in the calculated thickness. Similarly, for internal curvatures, the sag, s, at point x on the surface would have a maximum estimation error of δn_g_s. For the range of index errors shown in Fig. 3(g), this results in a maximum error of approximately −5 µm per millimeter of thickness; and <0.5% radius error, which can be verified with the radius equation $r\,=\frac{{s}^{2}\,+\,{x}^{2}}{2s}$. While these geometric errors may compound with the number of glass elements, our results showed no apparent correlation for at least up to 2 elements.

As previously mentioned, interrogation of optical elements using near-infrared light tended to overestimate Abbe numbers. This is likely because glass materials are less dispersive at these wavelengths as compared to the visible regime. Glass material identification accuracy may be improved by further constraining the glass material search-space to specific manufacturer catalogs based on prior information. For example, when characterizing Zeiss lenses, constraining glass materials to the Schott (Zeiss subsidiary) catalog would reduce the set of possible solutions by an order of magnitude. Here, only prior information about chromaticity in the visible wavelength range was leveraged to optimize the characterized model (Supplementary Fig. S4). Alternatively, RCM measurements for glass material identification may be performed at wavelengths in which refractive index differences dominate the measurement noise. As an example, this may be achieved using a supercontinuum source and performing measurements and ray-tracing simulations at wavelengths in both the visible and near-infrared ranges.

## Conclusion

We presented methods for non-contact characterization of compound optical elements to enable modeling of optical aberrations and optimization of system performance. Our combination of LCI and RCM measurements with computational ray-tracing was validated on commercial achromatic doublet lenses and a multi-element scan lens. Our approach for glass identification overcomes fundamental limitations in previously described methods for measuring material optical properties and may be directly extended to arbitrary surface geometries. System performance is robust and may be customized to specific applications and achieve desired measurement accuracies by tailoring the illumination and collection NA and wavelength. We believe our method will broadly impact optical imaging in research, by enabling design optimization when integrating commercial lenses, as well as commercial optical manufacturing by providing non-contact, real-time feedback on tolerances and performance.

## Methods

LCI and RCM used a superluminescent diode source (MT-850-HP, Superlum Diodes Ltd., Ireland) with 830 nm center wavelength and 140 nm full-width at half-maximum bandwidth. The LUT was mounted using a custom adapter and translated using a two-axis x-y stage (MLS203-2, Thorlabs Inc., USA) and one-axis z-stage (MTS25-Z8, Thorlabs Inc., USA).

### RCM acquisition

RCM measurements were performed by closing a pupil in the reference arm and only detecting signal from the sample arm. To find the focus at a particular surface, the LUT was first manually translated for coarse positioning until the detected signal was near maximum. The z-axis stage was then stepped electronically through ±40 µm around the manually positioned focus. The detected signal at the center wavelength was then used to plot the confocal axial response. Finally, a weighted mean localization was performed to determine the position of the focal plane (Fig. 3(a)).

### LUT centration

The LUT was mounted on a kinematic and aligned to minimize tilt relative to the illumination beam under LCI guidance. An initial LCI acquisition of LUT surface cross-sections followed by sphere-fitting were performed to determine decentration. The initial acquisition positions were then updated to center the LUT relative to the illumination beam, and the unknown surface was brought into focus before performing subsequent curvature measurements.

### LCI acquisition and processing

The reference arm pupil and detector exposure time were adjusted to maximize signal and fringe visibility for each surface. A background spectrum and x-y cross-sections of the LUT surface were acquired automatically. The processing code included background subtraction followed by spectral-apodization using a Hanning window to minimize ringing artifacts. Numerical dispersion compensation was performed^46^ and LCI cross-sections were segmented by taking the peak along each axial profile. The segmented data were then transformed from pixel units to physical units before sphere-fitting to obtain the radius of curvature.

## Supplementary information

Supplementary material

## Data Availability

All data and simulation/processing code are available upon request.
