# Supplementary material for: Non-contact characterization of compound optical elements using reflectance confocal microscopy, low-coherence interferometry, and computational ray-tracing
Source: Sci Rep. 2019 Nov 19;9:17111. doi: 10.1038/s41598-019-53369-x (PMC6864052; doi:10.1038/s41598-019-53369-x)
Supplement: Supplementary file 1 — Supplementary material [file 41598_2019_53369_MOESM1_ESM.pdf]

Non-contact characterization of compound optical elements using reflectance confocal microscopy, low-coherence interferometry, and computational ray-tracing

Supplementary Information

Mohamed T. El-Haddad* and Yuankai K. Tao*

Department of Biomedical Engineering, Vanderbilt University, Nashville, TN, USA

Correspondence should be addressed to M.T.H. (mtarek16@gmail.com) and

Y.K.T. (yuankai.tao@vanderbilt.edu)

Abstract

Advances in microscopy have enabled us to see at unprecedented depths and resolutions, even breaking the diffraction-limit by several fold. These improvements have come at the expense of system complexity with microscopes routinely employing multiple objective lenses and custom optical relays. Optimal system design is paramount for imaging performance, but research systems are limited by the use of commercial components because optical prescriptions are often inaccessible. System performance can be further degraded when these components are implemented in nonstandard configurations outside of manufacturer specifications. Here, we describe a method for characterization of compound optical elements including curvatures, material and air-gap thicknesses, and glass types. We present validation data for doublets and a commercial broadband scan lens. Our method is both non-contact and non-destructive, and we believe it addresses a unique gap in optical design that may be extended to broad applications in both research and industrial manufacturing.

Materials and Methods

Interferometer design and radius measurement repeatability

In the imaging system design (Fig. 1 (a)) the single-mode fiber core in the fiber-coupler acted as a pinhole to maintain confocal detection, and an uneven split was employed to maximize collection efficiency. The SNR was mainly limited by the available dynamic range of the CMOS sensor, but the exposure time was adjusted per acquisition to maximize SNR for each surface. The reference arm power was adjusted by a pupil to maximize fringe visibility. This design also allowed performance comparison between fiber-based and free space-based interferometers. While the accuracy was comparable, the measurement repeatability was significantly improved when using the free space interferometer (Fig. S1). This is due to the stronger mechanical coupling between the reference and sample arms in the free-space design compared to the fiber-based one.

SNR thresholding for evaluating curved surfaces

As outlined in the manuscript, a -27.5 dB threshold was applied to the sampled surface points prior to sphere fitting to obtain the radius of curvature. Figure S2 shows the measurement error from the reference sphere before and after SNR thresholding. It can be seen that thresholding enhances the measurement accuracy regardless of the sampling density.

The empirically determined SNR threshold limited the points used in the sphere fit for the reference sphere to within < 1 mm decenter (Fig. S3). The surface normal of the reference sphere at 1 mm makes an angle that is approximately half the NA of the collection optics. Therefore, SNR thresholding essentially rejects points corresponding to surface tilts beyond NA/2. This is in agreement with previously published recommendations in confocal profilometry where allowable tilts were to be limited to NA/2 to minimize measurement errors[1]. When imaging surfaces of unknown curvatures, SNR thresholding is advantageous as a simple method for limiting the collected angles.

Aspheric fit for internal surfaces

LCI profiles of internal surface were first acquired by sampling two orthogonal cross-sections similar to external surfaces, and the output image was fit to a sphere to obtain the distorted radius of curvature. Spherical surfaces representing the distorted internal surface and its upstream surfaces were then generated over a XY grid, separated axially by the measured geometric thicknesses. Finally, chief rays were propagated through all the surfaces over the entire grid based on the identified glass material properties, and the final surface image was corrected for distortions due to refraction and path-length scaling. However, since distortion-correction was only performed along the forward path, the final surface was aspheric. Direct sphere fitting was thus not suitable, and an aspheric fit was performed instead as explained in the manuscript. The following tables show representative data from 4 doublet lenses comparing variability in the distortion-corrected radius obtained with spherical vs aspheric fitting, with the grid size varied from 0.2 to 0.8 of the respective lens diameters. It can be seen that the aspheric fit provides significantly more consistent values for the radius of curvature compared to the spherical fit.

An alternative approach would be to correct for the forward and reflected paths to obtain a more accurate representation of the spherical surface. However, since the surface curvature is unknown, the curvature must be iteratively optimized until the simulated distorted surface image matches the imaged surface.

References

1. Aguilar, J. F. & Mendez, E. R. On the limitations of the confocal scanning optical microscope as a profilometer. *Journal of Modern Optics* **42**, 1785–1794 (1995).

Figures and Tables

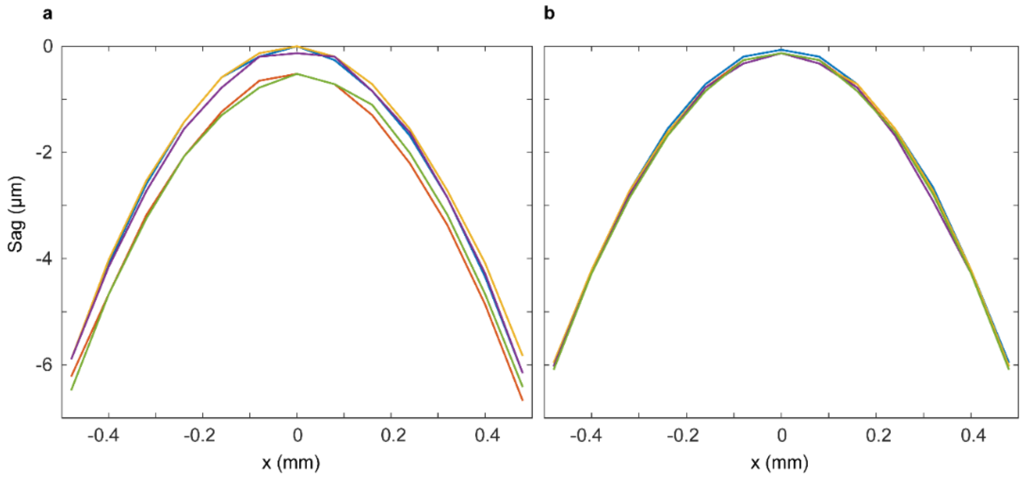

Figure S1 – Surface sag plots from repeated acquisitions (n = 5) of a lens surface profile using (a) a fiber- and (b) free-space based Michelson interferometer.

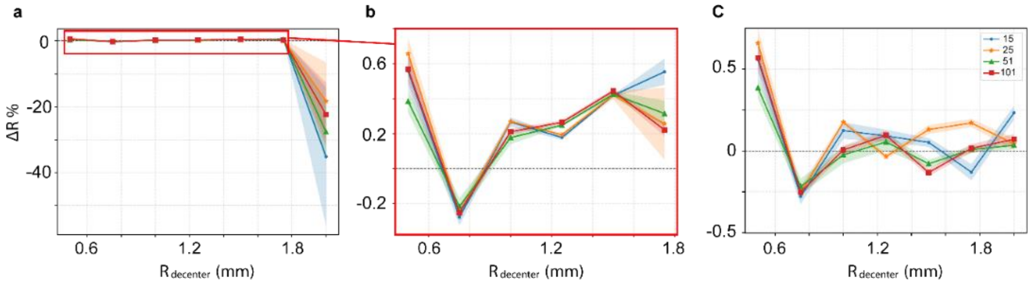

Figure S2 – Effect of SNR thresholding on radius measurement accuracy. (a) The error increases significantly at 2 mm decenter due to loss of signal. (b) A magnified view of the percent error in the red box in (a) before, and (c) after SNR thresholding.

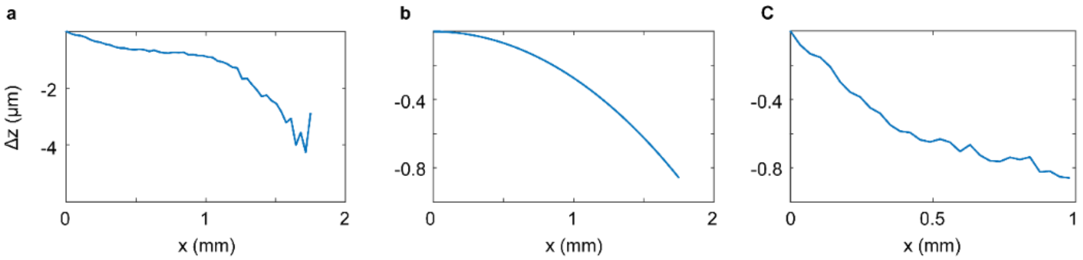

Figure S3 – Deviation between measured and expected surface sag of the reference sphere. (a) Deviation at sampling decenter out to 1.8 mm shows a rapid increase in error beyond 1 mm. (b) Difference in sag due to manufacturing tolerance in the reference sphere diameter over the acquisition range is an order of magnitude

less than the measurement errors shown in (a), indicating that the latter is the dominant source of error. (c) The retained points after SNR thresholding lie within 1 mm decenter.

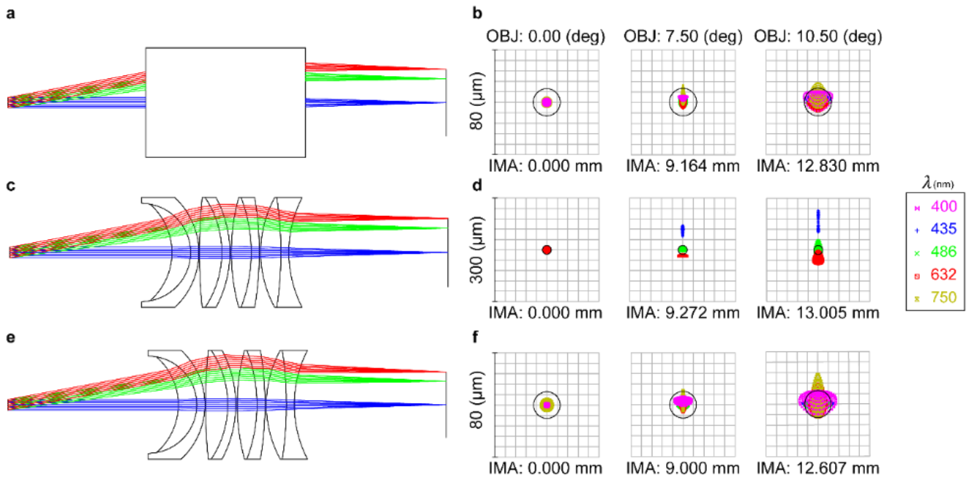

Figure S4 – Ray-trace and spot diagram comparison of optical performance. (a), (b) Manufacturer black-box lens model performance for 0, 7.5, and 10.5 degree object space field angles; and corresponding performance for empirically measured prescription data (c), (d) before, and (e), (f) after optimization of glass materials

Interferometer Design	Mean Radius (mm)	Standard Deviation (mm (%))	Range (mm (%))
**Fiber**	18.899	0.081 (0.43%)	0.222 (1.17%)
**Free-space**	18.893	0.005 (0.03%)	0.012 (0.06%)

Table S1 – Comparison between measurement errors for the surface acquisitions in Figure S1 (n = 5)

Lens Model	Radius (spec mm)	Fit type	Grid size / lens diameter			
			0.2	0.4	0.6	0.8
**AC127-025-A**	R2 (10.6)	S	10.674	10.511	10.183	9.545
		A	10.725	10.725	10.729	10.726
	R3 (68.1)	S	68.852	69.421	70.449	72.068

		A	68.669	68.664	68.668	68.619
**AC254-200-A**	R2 (87.6)	S	87.435	87.325	87.136	86.860
		A	87.472	87.472	87.471	87.471
	R3 (291.1)	S	292.298	292.195	292.023	291.783
		A	292.332	292.333	292.332	292.333
**ACN254-050-A**	R2 (32.5)	S	31.830	31.303	30.252	28.088
		A	31.992	31.991	31.984	32.011
	R3 (189.2)	S	185.027	187.011	190.543	196.050
		A	184.386	184.394	184.421	184.127
**ACN254-100-A**	R2 (49.9)	S	50.520	50.334	50.005	49.495
		A	50.580	50.579	50.579	50.576
	R3 (600)	S	589.157	594.478	603.679	617.300
		A	587.419	587.421	587.418	587.431

Table S2 – Comparison between spherical (S) and aspheric (A) fitting for measurement of radii of internal surfaces.

**Surface #**	**Radius**	**Thickness**	**Material**	**Phase index**	**Abbe Number**
**1**	-24.558	7.099	BAM23	1.5959	40.26
**2**	-18.759	3.128	L-LAH84P	1.7971	40.29
**3**	-28.697	0.240	Air		
**4**	215.473	8.504	K-PFK80 (M)	1.4904	80.66
**5**	-37.975	3.072	S-YGH52	1.7743	50.00
**6**	-44.534	0.560	Air		
**7**	79.883	7.577	K-PFK85 (M)	1.4792	85.07
**8**	-58.968	3.078	D-LAF50	1.7620	49.60
**9**	-127.415	0.540	Air		
**10**	44.102	7.692	K-PFK85 (M)	1.4792	85.07
**11**	-142.075	3.021	MC-NBFD135	1.7935	40.92
**12**	46.196				

Table S3 – Characterized lens prescription before glass material optimization.

Surface #	Radius	Thickness	Material	Phase index	Abbe Number
**1**	-24.558	7.099	PBM3	1.6007	36.99
**2**	-18.759	3.128	LAH59	1.8026	46.63
**3**	-28.697	0.240	Air		
**4**	215.473	8.504	J-FKH1	1.4930	82.57
**5**	-37.975	3.072	NBFD11	1.7722	43.93
**6**	-44.534	0.560	Air		
**7**	79.883	7.577	K-PFK85	1.4811	85.19
**8**	-58.968	3.078	M-TAF401	1.7611	47.17
**9**	-127.415	0.540	Air		
**10**	44.102	7.692	K-PFK85 (M)	1.4792	85.07
**11**	-142.075	3.021	H-ZLAF1	1.7878	44.28
**12**	46.196				

Table S4 – Characterized lens prescription after glass material optimization.

Surface #	Glass material	Phase index	Abbe number
**1**	N-BAF4	1.59516	43.72
	N-BAF4	1.59516	43.72
	BAM23	1.59593	40.26
	BAM5	1.59784	49.19
	H-BAF6	1.59800	46.22
	N-BAF52	1.59863	46.60
	PBM3	1.60067	36.99
**2**	LAM66	1.78407	34.97
	L-LAH84	1.79335	40.55
	P-LASF50	1.79350	40.46
	P-LASF51	1.79503	40.92
	L-LAH84P	1.79710	40.29
	S-LAH54	1.80147	44.36
	LAH59	1.80258	46.63
**4**	N-FK5	1.48176	70.40
	FSL5	1.48178	70.21
	K-PFK80(M)	1.49042	80.66
	Q-FK01S	1.49163	81.67
	N-PK52A	1.49215	81.61
	J-FKH1	1.49303	82.57

**5**	NBFD11	1.77217	43.93
	N-LAF33	1.77222	44.05
	S-YGH52	1.77425	50.00
	J-LASF014	1.77515	47.35
	K-VC179(M)	1.78006	41.85
	N-LAF32	1.78115	45.53
**7**	K-PFK85(M)	1.47925	85.07
	K-PFK85	1.48111	85.19
	N-FK51A	1.48195	84.47
**8**	J-LASFH2	1.75416	46.78
	L-LAH87	1.75784	47.40
	N-LAF34	1.76038	49.62
	M-TAF401	1.76113	47.17
	D-LAF50	1.76198	49.60
**10**	K-PFK85(M)	1.47925	85.07
	K-PFK85	1.48111	85.19
	N-FK51A	1.48195	84.47
**11**	H-ZLAF1	1.78778	44.28
	J-LASFH6	1.78841	33.34
	J-LASF013	1.78907	39.61
	N-LASF44	1.79089	46.50
	NBFD13	1.79117	40.73
	K-VC181(M)	1.79137	41.05
	MC-NBFD135	1.79345	40.92
	D-ZLAF52LA	1.79502	41.00
	L-LAH84P	1.79710	40.29

Table S5 – Glass candidates selected for each material in the lens prescription. Bolded names are the initially identified materials before optimization.

#	Type	Int1	Int2	Hx	Hy	Px	Py	Target	Weight	Value	Contrib
**1**	CNPX	18	1	0.500	0.500	0.000	5.000	0.000	0.000	6.282	0.000
**2**	CNPX	18	4	0.500	0.500	0.000	5.000	0.000	0.000	6.283	0.000
**3**	CNPX	18	2	0.500	0.500	0.000	5.000	0.000	0.000	6.281	0.000
**4**	CNPX	18	3	0.500	0.500	0.000	5.000	0.000	0.000	6.282	0.000

**5**	CNPX	18	5	0.500	0.500	0.000	5.000	0.000	0.000	6.284	0.000
**6**	DIFF	2	2	0.000	0.000	0.000	0.000	0.000	1.000	0.000	0.000
**7**	DIFF	2	3	0.000	0.000	0.000	0.000	0.000	1.000	0.002	50.761
**8**	DIFF	2	4	0.000	0.000	0.000	0.000	0.000	1.000	0.001	23.736
**9**	DIFF	2	5	0.000	0.000	0.000	0.000	0.000	1.000	- 0.001	19.039
**10**	RSCE	1	0	0.000	0.000	0.000	0.000	0.000	1.000	0.001	6.464

Table S6 – Merit function used in Zemax to minimize lateral chromatic aberration in the visible wavelength range.
